# Active transport of rhodamine 123 by the human multidrug transporter P‐glycoprotein involves two independent outer gates

**DOI:** 10.1002/prp2.572

**Published:** 2020-03-31

**Authors:** Fauzia Nasim, Diethart Schmid, Gergely Szakács, Azmat Sohail, Harald H. Sitte, Peter Chiba, Thomas Stockner

**Affiliations:** ^1^ Institute of Medical Chemistry Center for Pathobiochemistry and Genetics Medical University of Vienna Vienna Austria; ^2^ Institute of Physiology Center for Physiology and Pharmacology Medical University of Vienna Vienna Austria; ^3^ Institute of Cancer Research Medical University of Vienna Vienna Austria; ^4^ Institute of Pharmacology Center for Physiology and Pharmacology Medical University of Vienna Vienna Austria

**Keywords:** ABC transporter, ABCB1, functional biology, multidrug transporter, outer gates, P‐glycoprotein

## Abstract

Human P‐glycoprotein (P‐gp) is a multispecific drug‐efflux transporter, which plays an important role in drug resistance and drug disposition. Recent cryo‐electron microscopy structures confirmed its rotationally symmetric architecture, which allows dual interaction with ATP and substrates. We here report the existence of two distinct, symmetry‐related outer gates. Experiments were aided by availability of the X‐ray structure of a homodimeric eukaryotic homolog of P‐gp from red alga (CmABCB1), which defined the role of an apical tyrosine residue (Y358) in outer gate formation. We mutated analogous tyrosine residues in each half of the human full‐length transporter (Y310, Y953) to alanine. These mutants were introduced in engineered transporters which bind rhodamine 123 in one of two symmetry‐related binding modes only. Outer gate dysfunction was detected by a loss of active transport characteristics, while these mutants retained the ability for outward downhill transport. Our data demonstrate that symmetric tyrosine residues Y310 and Y953 are involved in formation of two distinct symmetry‐related outer gates, which operate contingent on the rhodamine 123 binding mode. Hence, the rotationally symmetric architecture of P‐gp, which determines duality in ATP binding and rhodamine 123 interaction, also forms the basis for the existence of two independently operating outer gates.

## Graphical abstract

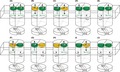

**Abbreviations**
ABC: ATP‐binding cassette
CEM: cryo‐electron microscopy
HEK293: human embryonic kidney 293 cells
MDRs: multidrug resistance transporters
NBD: nucleotide binding domain
NBS: nucleotide binding site
P‐gp: P‐glycoprotein
rh123: rhodamine 123
TM: transmembrane helix
TMDs: transmembrane domains
TRQ: tariquidar
ZTE: zero‐trans efflux

## 1 INTRODUCTION

P‐glycoprotein (P‐gp), a member of the human ATP‐binding cassette (ABC) family, is a multispecific drug‐efflux transporter (MDR). A key physiological role is protection of the organism from environmental toxins.^1^ Many drugs that act on intracellular target structures have been identified as substrates. They are intercepted at the level of the plasma membrane and transported back to the cell exterior. Consequently, P‐gp is a major cause for treatment failure.^2^ Equally important is its role in drug‐disposition and drug‐drug interactions.^3^, ^4^

Recently, an ATP‐bound outward‐facing structure of human P‐gp^5^ and a structure of nucleotide‐free human P‐gp in the presence of the substrate taxol^6^ were resolved by cryo‐electron microscopy (CEM). These structures confirm the anticipated twofold rotational (also referred to as cyclic twofold or C2) symmetry of the transporter. In the first structure, two molecules of ATP are found in two symmetry‐related nucleotide binding sites. While for ATP, the existence of two binding sites has long been appreciated from a number of higher resolution structures of other ABC transporters, the interaction of P‐gp with substrates has remained a matter of debate. The second CEM structure of human P‐gp^6^ provides a first structural example of P‐gp in the presence of one of its substrates.

Using photolabeling and high‐resolution mass spectrometry, our group previously provided biochemical evidence for dual symmetry‐related interaction of P‐gp with propafenone analogs.^7^ In addition, interaction with the paradigmatic substrates rhodamine 123 (rh123), vinblastine, and verapamil was shown to occur in two symmetry‐related modes.^8^

Data presented in this manuscript provide information about the process of outer gating. Active transport by P‐gp is thought to require the alternating opening and closure of inner and outer gates, which are formed by interaction of residues on the cytoplasmic and extracellular side of the substrate binding pocket.^9^ As ATP binding and substrate binding occur in symmetry‐related manner, the questions arose, if (a) the substrate rh123 would leave the central pocket via a single exit path, as proposed by Al‐Shawi,^10^ or alternatively, if transport would rely on two distinct exit paths and (b) in the case of the existence of two distinct exit paths, if use of these exit paths would be determined by the binding mode, which the substrate rh123 adopts within the central binding pocket of the transporter.

Several residues were shown to contribute to the formation of an outer gate in a eukaryotic homolog of human P‐gp from *Cyanidioschyzon merolae* (CmABCB1; PDB ID: http://www.rcsb.org/pdb/search/structidSearch.do?structureId=3WMG).^11^ CmABCB1 is a half‐transporter, which requires homodimerization for function, while human P‐gp is a full‐length transporter, in which a single gene encodes for the two transmembrane and the two nucleotide binding domains. Tyrosine residues Y310 and Y953 in human P‐gp correspond to residues Y358 and Y358′ in the two identical monomers of the homodimeric red alga transporter. The Y310A and Y953A mutants were generated and functionally characterized in transport assays. In order to investigate, if gating would be contingent on the substrate binding mode, rh123 binding had to be restricted to one of the two possible binding modes. Selection of one or the other of these binding modes was brought about as described earlier.^8^ Briefly, selection was based on charge repulsion between the permanent positive charge of rh123 on the one hand and positively charged arginine residues, introduced in symmetric positions 132 (TM2) and 773 (TM8) of human P‐gp, on the other hand. Tariquidar (XR9576) (TRQ) is a potent third‐generation inhibitor of P‐gp, which has the ability to block active efflux with an IC_50_ value in the low nanomolar range.^12^ It was used as a specific inhibitor of human P‐gp in order to prove that rh123 transport in the mutants was indeed transporter mutant mediated. Our study provides functional evidence that (a) the substrate rh123 exits from the central binding pocket via two symmetry‐related outer gates and (b) use of each of these individual outer gates is governed by the binding mode, which the substrate adopts in the central cavity of human P‐gp prior to its release into the extracellular space.

## 2 MATERIALS AND METHODS

### 2.1 Materials

Rh123, TRQ, Dulbecco's Modified Eagle's Medium (DMEM, high glucose), and fetal bovine serum were purchased from Sigma‐Aldrich. HEPES was obtained from Roth.

### 2.2 Human embryonic kidney 293 cells

HEK293 cells, in which endogenous P‐gp was knocked down by lentiviral transduction with two siRNAs directed against the 3′ UTR of the MDR1 gene,^13^ were grown in DMEM supplemented with 10% fetal bovine serum under standard culture conditions. Cells were harvested, centrifuged at 500*g*, and washed once with ice cold phosphate‐buffered saline (PBS) prior to experiments.

### 2.3 Vector constructs and transfection

Mutants were generated in a gateway compatible vector system in the pENTR4 entry vector (Invitrogen). The forward and reverse primers (Microsynth) used in this study are listed in Table 1. The gateway technology,^14^ which exploits recombinatorial properties of bacteriophage lambda, was used to transfer the ABCB1 insert into the proprietary gateway compatible pCEP4d destination vector as described.^8^ Authenticity of all mutants was confirmed by sequencing. Wild‐type and mutant P‐gp was transfected into HEK293 cells as described previously.^13^

**Table 1:** List of forward and reverse primers used for generation of mutants

Mutations	Primers	
Y310A	Forward	5′‐tgctttcctgctgatctatgcatctgctgctctggccttc‐3′
	Reverse	5′‐gaaggccagagcagcagatgcatagatcagcaggaaagca‐3′
Q725L	Forward	5′‐ccattataaatggaggcctgctaccagcatttgcaataatatt‐3′
	Reverse	5′‐aatattattgcaaatgctggtagcaggcctccatttataatgg‐3′
Y953A	Forward	5′‐cccaggcaatgatgtatttttccgctgctggatgtttccggtt‐3′
	Reverse	5′‐aaccggaaacatccagcagcggaaaaatacatcattgcctggg‐3′
E556Q	Forward	5′‐caagatcctcctgctggatcaggccacgtc‐3′
	Reverse	5′‐gacgtggcctgatccagcaggaggatcttg‐3′
Q132R	Forward	5′‐gtgctggttgctgcttacattagagtttcattttggtgcctggca‐3′
	Reverse	5′‐tgccaggcaccaaaatgaaactctaatgtaagcagcaaccagcac‐3′
Q773R	Forward	5′gcccttggaattatttcttttattacatttttccttagaggtttcacatttggcaaagc‐3′
	Reverse	5′‐cgggaaccttaataaagaaaataatgtaaaaaggaatctccaaagtgtaaaccgtttcg‐3′
K433M	Forward	5′‐cagtggctgtgggatgagcacaacagtcc‐3′
	Reverse	5′‐ggactgttgtgctcatcccacagccactg‐3′
K1076M	Forward	5′‐cagtggctgtgggatgagcacagtggtcc‐3′
	Reverse	5′‐ggaccactgtgctcatcccacagccactg‐3′

### 2.4 Visualization of residues in CmABCB1 and human P‐gp

The location of residues is indicated in the X‐ray structure of CmABCB1 (PDB ID: http://www.rcsb.org/pdb/search/structidSearch.do?structureId=3WMG)^11^ and the cryo‐electron microscopy structure of nanodisc reconstituted human P‐gp (PDB ID: http://www.rcsb.org/pdb/search/structidSearch.do?structureId=6QEX)^6^ using the software program Visual Molecular Dynamics (VMD).^15^

### 2.5 Surface expression of wild type and mutants

Surface expression of P‐gp was assessed using the MRK16 monoclonal antibody (final concentration 5 µg/mL, Kamiya Biomedical Company) and approximately 5 × 10^5^ cells per data point. IgG2A (2.5 μg/mL) was used as the control antibody. Cells were incubated for 30 minutes, washed with ice‐cold phosphate‐buffered saline (PBS), and centrifuged at 500*g*. After resuspension, cells were incubated with the secondary fluorescein‐isothiocyanate (FITC)‐labeled goat anti‐mouse antibody (12.5 μg/mL) for 30 min on ice water in the dark. Cells were then washed with PBS, resuspended, and cellular fluorescence was measured using a flow cytometer (FACSCalibur, Becton Dickinson). The excitation wavelength was 488 nm and emission was monitored with a 530/30 nm band‐pass filter (FL1) as described.^13^

### 2.6 Rh123 transport assays

Rh123 steady‐state accumulation and efflux were measured as follows: Cells (0.75 × 10^6^/mL) were incubated in DMEM (pH 7.4) containing 25 mmol/L HEPES at 37°C and rh123 at a concentration of 1 µmol/L. After 20 minutes, a steady state of cellular loading was reached (Figure S1). Cells were chilled on ice‐water, centrifuged, and washed twice with medium. Cell pellets were then resuspended in 37°C HEPES‐buffered DMEM (HBDMEM), pH 7.4, and steady‐state accumulation levels were determined by flow cytometry. The decrease in cellular fluorescence was monitored continuously over 5 minutes. Following resuspension of cells in 37°C medium rh123 efflux requires about 20‐30 seconds for attaining exponential efflux characteristics. Therefore, data points acquired within the first 30 seconds were excluded from the exponential fits. First‐order rate constants of efflux were determined as described previously.^13^

### 2.7 TRQ inhibition assays

Cells were preloaded with rh123 at a final concentration of 1 µmol/L in HBDMEM, pH 7.4, at 37°C in the absence and presence of eight different concentrations of TRQ (1:3 serial dilutions starting from 405 nmol/L) until a steady‐state level of loading was reached. Cells were then washed free of Rh123 and resuspended in HBDMEM, pH 7.4, containing identical concentrations of TRQ. Efflux was monitored continuously over a time period of 5 minutes at a temperature of 37°C. Transport rates (*k*) were plotted as a function of inhibitor concentration and IC_50_ values were calculated by fitting hyperbolic concentration response curves to the data points.^16^

### 2.8 Kinetic modeling

The time course of substrate uptake and efflux in HEK293 cells expressing wild‐type ABCB1 were mathematically simulated based on a previously published kinetic model of the ABCB1 transport cycle^17^ with minor modifications. In particular, substrate membrane permeation between the interstitial space and a cellular accumulation space were taken into account. The cytosolic volume of a HEK293 cell was defined as the accumulation space. The geometry of the cell was assumed to be spherical with a diameter of 13 µm and a corresponding volume of 1150 femtoliters. Substrate uptake and efflux in cells expressing wild‐type P‐gp and transporter mutants were simulated. For facilitators, the marked decrease in outer substrate affinity, which is a feature of ATP‐driven active transporters, was eliminated. Substrate uptake and efflux via hybrid active/facilitative P‐gp were simulated by combining the two transport components so that they access an identical accumulation space. Time‐dependent changes in state occupancies, as well as cellular substrate concentration were evaluated by numeric integration of the resulting system of differential equations using the Systems Biology Toolbox^18^ and MATLAB 2012a (Math works).

### 2.9 Statistical analysis

Data were analyzed using the GraphPad prism software (GraphPad Software, Inc). One‐way ANOVA and Bonferroni post‐hoc analysis were used for comparison of groups. The levels of statistical significance are as follows: *P < .05; **P < .01; ***P < .001.

## 3 RESULTS

### 3.1 Outer gate residues in red alga CmABCB1 and their counterparts in human P‐gp

**Figure 1:**
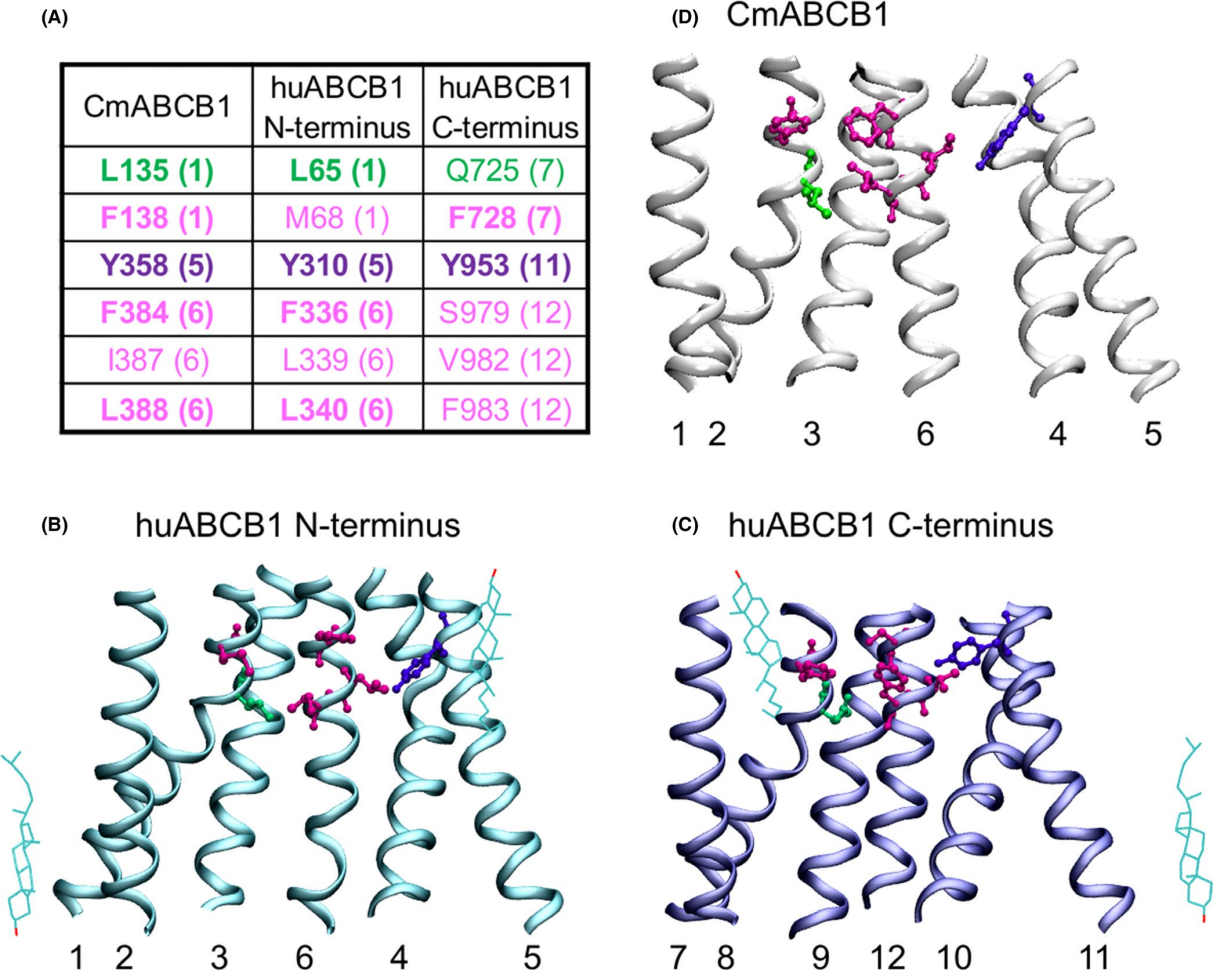
(A) Residues identified as forming the outer gate in CmABCB1 and aligning residues in the N‐ and C‐terminus of human P‐gp are shown in purple (mutated in this study) and magenta. Residue Q725, which can substitute for tyrosine Y310 in forming a functional outer gate, as well as aligning residues in the CmABCB1 monomer and the N‐terminus of human P‐gp are shown in green. Numbers in parentheses refer to transmembrane helices, in which these residues are found. Matching amino acid residues are shown in bold. (B) Transmembrane region of the CmABCB1 X‐ray structure^11^ (PDB ID: http://www.rcsb.org/pdb/search/structidSearch.do?structureId=3WMG) in a side view. The extracellular side is up. Residues are shown in licorice rendering in the same color as in A. (C) Transmembrane region of the N‐terminus of human P‐gp^6^ (PDB‐ID: 6QEX) in a side view. Same color and rendering of residues as in B. (D) Transmembrane region of the C‐terminus of human P‐gp. Same color and rendering as in B and C. Hydroxyl groups of structurally resolved cholesterol molecules in C and D delineate the inner and outer membrane leaflet water interface planes, which run in parallel to the viewing axis

Kodan and co‐authors identified amino acid residues, which contribute to outer gate formation in a eukaryotic homolog of human P‐gp from red alga (CmABCB1, PDB ID: http://www.rcsb.org/pdb/search/structidSearch.do?structureId=3WMG).^11^ Among them only two tyrosine residues, which are located in the apex of the central cavity, are conserved between Cyanidioschyzon merolae and human P‐gp (Y358, Y358′ in the CmABCB1 homodimer,Y310, Y953 in the N‐ and C‐terminal half of the human full‐length transporter) (Figure 1A). The membrane‐spanning portion of one monomer of the homodimeric CmABCB1 X‐ray structure is shown in a side view with gating residues highlighted in magenta/purple (Figure 1B). Analogous residues in the N‐ and C‐terminus of human P‐gp are shown in Figure 1C and D, respectively, in the CEM‐structure of P‐gp^6^ (PDB ID: http://www.rcsb.org/pdb/search/structidSearch.do?structureId=6QEX) for visualization.

### 3.2 Identification of transporter mutants with a dysfunctional outer gate

Active transport is considered to rely on the alternating opening and closing of inner and outer gates.^9^, ^19^, ^20^ When the outer gate is rendered deficient by mutation, these mutants can be identified by their loss of active transport characteristics. In other words, these mutants are still able to transport rh123 along a concentration gradient (higher zero‐trans efflux rates than negative controls), but fail to decrease the free intracellular rh123 concentration below that on the outside.

Accordingly, we mutated tyrosine residues Y310 and Y953 to alanine and determined rh123 steady‐state accumulation and zero‐trans efflux rates in these two single mutants. The E556Q mutant (harboring a mutated catalytic glutamate in the Walker B motif of NBS1), mock transfected cells, and the Walker A double mutant K433M/K1076M^21^ served as negative controls. In these three negative controls, comparable steady‐state accumulation levels of approximately 1000 MFU/cell were found. When these negative controls were washed free of rh123 containing medium and resuspended in medium containing no rh123, the fluorescent substrate decreased at a rate of approximately 0.0005/s. This outward flux of rh123 is not P‐gp mediated.

**Figure 2:**
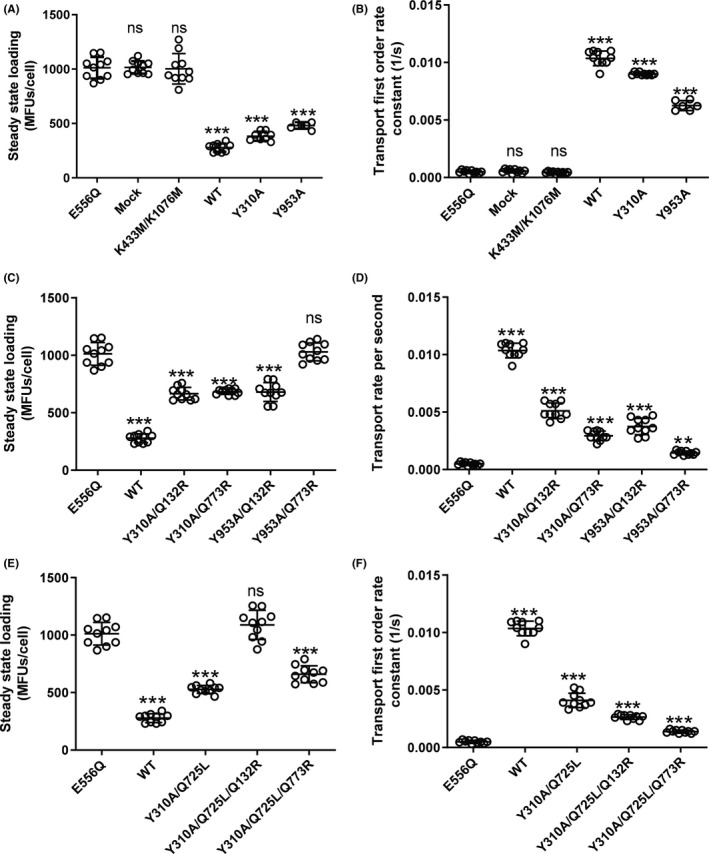
Steady‐state loading levels (A,C,E) and transport rates (B,D,F) in wild‐type P‐gp, mutants and negative controls. Data of 10 independent experiments are shown. Mutants are grouped in the following way: (A and B) Tyrosine mutants Y310A and Y953A in comparison with wild‐type (positive control) and the catalytic glutamate mutant E556Q, mock‐transfected cells and the Walker A lysine double mutant K433M/K1076M (negative controls). (C and D) Tyrosine mutants Y310A and Y953A introduced in the background of the binding‐mode selector mutants Q132R and Q773R. (E and F) Double mutant Y310A/Q725L in comparison with triple mutants Y310A/Q725L/Q132R and Y310A/Q725L/Q773R. Wild‐type and the catalytic glutamate mutant E556Q are shown for comparison. The central horizontal bar corresponds to the mean. Error bars refer to standard deviation. Statistical significance was determined by one‐way ANOVA using the Bonferroni multiple comparison post‐hoc test. Statistical significance levels are indicated by asterisks (***P < .001). ns, not statistically significant

The efflux rate observed in cells transfected with wild‐type P‐gp was approximately 20‐fold higher and resulted in steady‐state loading levels of about 250 MFU/cell. The two single mutants Y310A and Y953A retained the ability to decrease rh123 steady‐state loading levels below that on the outside, indicating retention of active transport characteristics in both mutants (Figure 2A and B).

**Figure 3:**
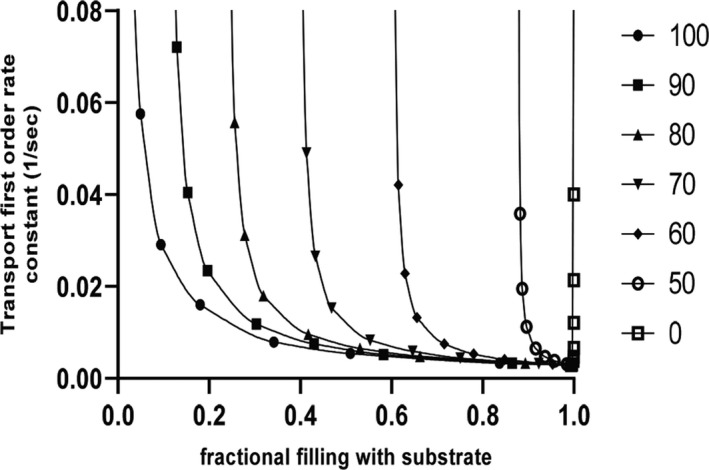
Relationship between fractional filling and transport rate (first‐order rate constant of transport) as predicted by kinetic modeling. Filled circles represent data points for wild‐type protein. The resulting curve is a rectangular hyperbola. The presence of a facilitative transport component, which would ensue from dysfunction of an outer gate, is predicted by the model to result in a right shift of the curves. Numbers to the right indicate the contribution (as a percentage of total flux) of the active transport component. When active transport is completely abolished (open squares), fractional filling has a value of 1.0 and becomes independent of the transport rate. The curve is a vertical line

This finding was not unexpected, as we considered the possibility that two functionally independent gates would be operative in P‐gp, and that these gates would function in dedicated manner with respect to the two symmetry‐related rh123 binding modes. Accordingly, wild‐type P‐gp would combine two active transport components, whereas in the mutants one of them would stay active, while the other would adopt characteristics of facilitative transport (ie, would be able to mediate downhill transport, but would fail to hold against a concentration gradient). In order to predict consequences of such a situation, a kinetic model was created in which relative contributions of the facilitative and active transport component were varied (Figure 3). The model predicts that mutants, in which one path would adopt facilitative characteristics, while the other would stay active, steady‐state loading levels would still be found decreased below those observed in negative controls.

Hence, we introduced tyrosine mutations Y310A and Y953A in a background, which would allow to select only one of the two rh123 binding modes (ie, mutants containing either the Q132R or the Q773R mutation). This experimental strategy was published previously.^8^ Accordingly, we generated four double mutants (Y310A/Q132R, Y310A/Q773R, Y953A/Q132R, and Y953A/Q773R), which were again characterized with respect to transport rates and rh123 steady‐state accumulation levels.

Results of these experiments are shown in Figure 2C and D. The Y953A/Q773R double mutant now showed steady‐state accumulation levels of substrate, which were indistinguishable from that in negative controls, while the rate for downhill transport of rh123 was significantly higher than that in negative controls. Thus, tyrosine 953 was confirmed to be involved in outer gating, when rh123 binding was restricted to mode 2 binding.^8^ In contrast, mutant Y953A/Q132R, in which rh123 was restricted to mode 1 binding, retained active transport characteristics.

### 3.3 Residue Q725 can substitute for the loss of the aromatic ring in the Y310A mutant and thus retain functionality of outer gate 1

**Figure 4:**
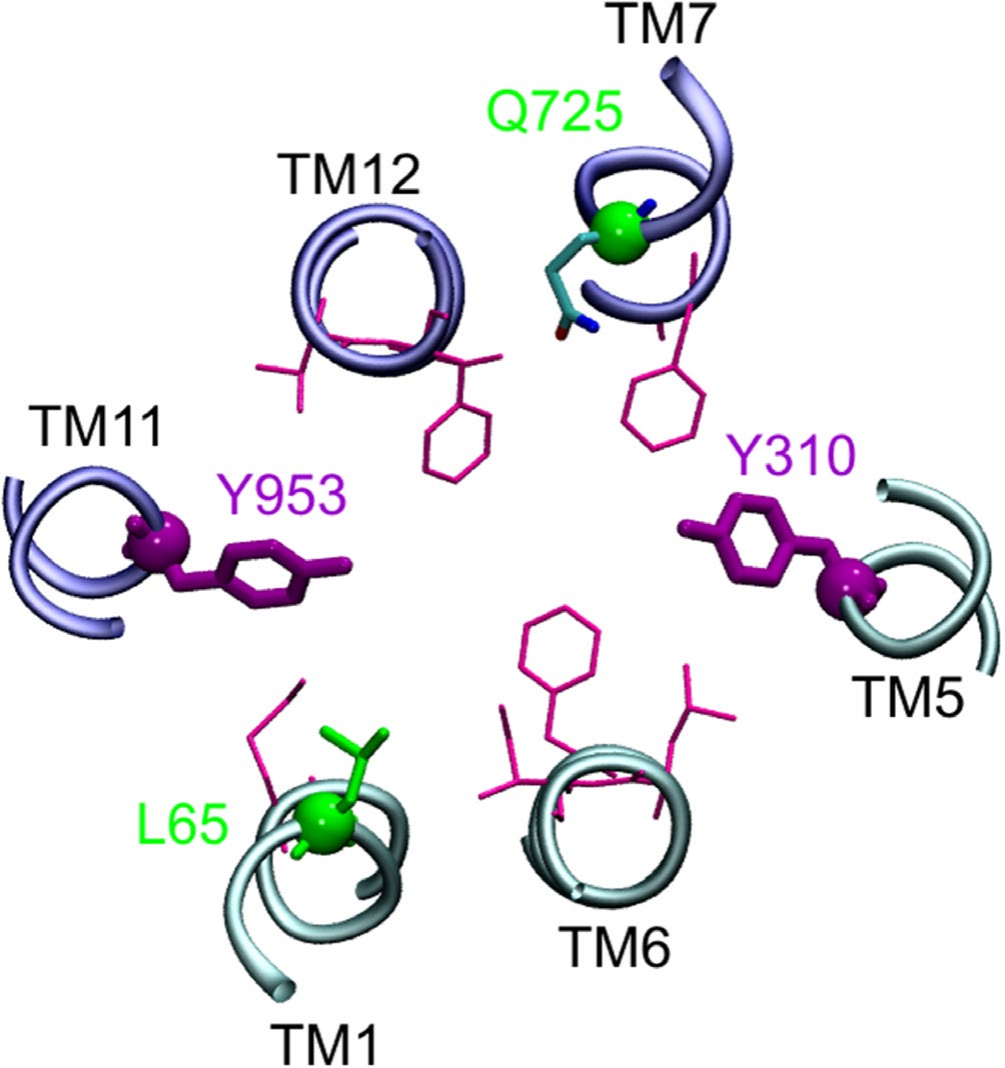
Position of residues from Figure 1 in a CEM structure of human P‐gp (PDB ID: http://www.rcsb.org/pdb/search/structidSearch.do?structureId=6QEX)^6^ viewed from the bottom. One to two helical turns of the respective transmembrane helices are shown. The N‐terminus is depicted in cyan (TMs 1, 6, 5), the C‐terminus in dark blue (TMs 11, 12, 7). Rendering of residues as in Figure 1. The distance between the polar side chains of residues Y310 and Q725 is below 7Å. When considering alternative rotamers of the amino acid side chains, distance between the two residues decreases further

The concept of the existence of two symmetry‐related outer gates would have predicted that the Y310A/Q132R mutant should also show a gate dysfunction. This however was not observed. A comparison of the X‐ray structure of CmABCB1^11^ and the CEM structure of human P‐gp^6^ revealed that leucine residue L135 in CmABCB1 (highlighted in green in Figure 1A conforms to a leucine residue in the N‐terminal half (L65) (Figure 1C) but to a polar glutamine residue in the C‐terminal half of human P‐gp (Q725) (Figure 1D). We thus considered the possibility that the presence of this polar glutamine residue may compensate for the loss of the hydroxyphenyl moiety of tyrosine Y310 in the Y310A mutant and that this would account for the failure to detect a gating deficiency in the Y310A/Q132R mutant. As shown in Figure 4, residues Y310 and Q725 are in close vicinity of each other. We thus generated the Y310A/Q725L double mutant and again introduced it in the background of the binding mode selector mutants Q132R and Q773R. Results of these experiments are shown in Figure 2E and F. We now found that the triple mutant Y310A/Q725L/Q132R, which allows rh123 binding in mode 1 only, also lost active transport characteristics. In contrast, the Y310A/Q725L/Q773R mutant retained the ability to decrease intracellular steady‐state concentrations of rh123 below that of negative controls. This means that it retained active transport characteristics.

**Figure 5:**
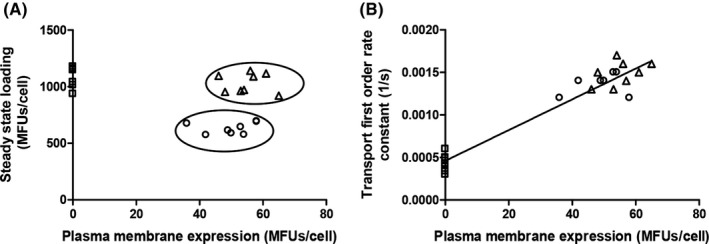
Steady‐state levels of rh123 accumulation (A) and transport rate (B) in the Y953A/Q773R mutant (triangles) and the Y310A/Q725L/Q773R (circles) as a function of plasma membrane expression. The transport rate of the two mutants is comparable and linearly dependent on protein expression. Rh123 efflux rates and steady‐state accumulation levels of the negative control are shown on the ordinate as squares. The Y953A/Q773R mutant is not able to decrease steady‐state loading, while the Y310A/Q725L/Q773R mutant is. The complete separation of the two populations is indicated by ovals in A

The Q773R mutation alone decreases rh123 transport to about 25% of wild‐type protein.^8^ Transport rates are further reduced by the introduction of the Y953A mutation. Low transport rates of the Y953A/Q773R were therefore considered to potentially account for a failure to detect a decrease in steady‐state loading levels in this double mutant. In order to exclude this possibility, we compared the Y953A/Q773R and the Y310A/Q725L/Q773R mutants with respect to their ability to decrease steady‐state loading levels of cells with rh123 (Figure 5A). Figure 5B shows that both mutants have comparable rh123 transport rates and P‐gp expression levels. While the Y310A/Q725L/Q773R mutant was able to significantly decrease steady‐state accumulation levels, the Y953A/Q773R mutant was not. This proves that the low transport rate in the Y953/Q773R mutant is not responsible for the inability to decrease rh123 steady‐state accumulation levels.

### 3.4 Rh123 transport in gating‐deficient mutants can be inhibited by TRQ

In order to rule out that P‐gp‐mediated efflux in mutants was leak transport, concentration response curves with the potent and specific P‐gp inhibitor TRQ were obtained. For all tyrosine mutants, a concentration‐dependent inhibition of zero‐trans efflux with a comparable range of TRQ IC_50_ values was found (Figure S3 and Table S1).

## 4 DISCUSSION

We previously found that rh123 binds to human P‐gp in two modes, which are related to each other by 180° rotational symmetry. This property of twofold interaction with rh123 is rooted in the rotationally symmetric architecture of the transporter, which has recently been confirmed by two CEM structures.^5^, ^6^ In the present study, we demonstrate that P‐gp harbors two independently operating, symmetry‐related outer gates. The rh123 binding mode determines which of the two gates is used by the substrate. Publication of the crystal structure of a multidrug transporter from *Cyanidioschyzon merolae* (CmABCB1) formed an important prerequisite for our studies. In this eukaryotic homolog of human P‐gp, candidate amino acid residues that contribute to outer gate formation were first identified in the X‐ray structure and subsequently their role in outer gating was confirmed by cytotoxicity assays. Mutation of tyrosine residue Y358 to alanine resulted in a complete loss of resistance to rhodamine 6G.^11^ CmABCB1 is a half‐transporter, which requires homodimerization for function. In contrast, human P‐gp is a full‐length transporter, in which all four domains are comprised in a single polypeptide chain. The two halves of P‐gp are therefore similar, while those of CmABCB1 are identical. Tyrosine residues Y358 and Y358′ in the CmABCB1 homodimer align with residue Y310 in the N‐terminus and Y953 in the C‐terminus of human P‐gp. The residue pair Y358/Y358′ in homodimeric CmABCB1 is the only pair, which matches a corresponding pair in human P‐gp (Y310/Y953) (Figure 1A). Individual mutation of residues Y310 and Y953 in human P‐gp to alanine was therefore expected to answer the question, if rh123 would leave the central drug‐binding cavity of P‐gp via a single exit path,^10^ or if evidence for two distinct symmetry‐related outer gates could be provided experimentally.

As discussed above, we previously showed that rh123 binds to human P‐gp in two modes, which are related to each other by 180° rotational symmetry.^8^ Binding in one of the two modes is favored over the other. The biochemically defined R‐ and H‐site of P‐gp^22^ likely relate to these two alternative substrate binding modes. We also showed that one of these binding modes can be deselected by introducing positively charged arginine residues in symmetric positions 132 (TM2) and 773 (TM8) of the transporter.^8^ This strategy exploits charge repulsion between the permanent positive charge of rh123 and these arginine residues. In the present study, tyrosine mutations Y310A and Y953A were introduced in a transporter background containing these binding‐mode selector mutations. Gating‐deficient mutants were subsequently identified in transport assays by their loss of active transport characteristics.

**Figure 6:**
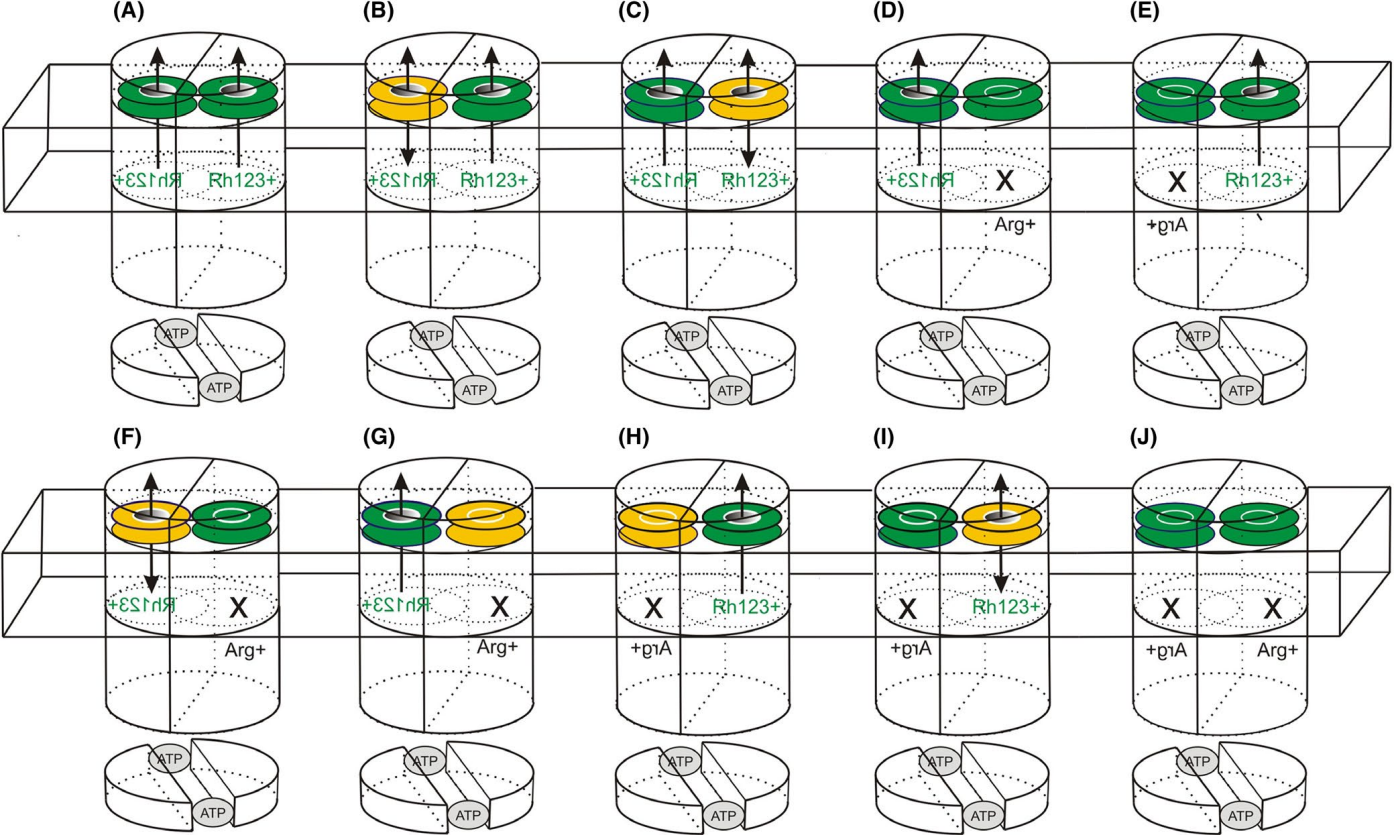
Schematic model of the outer gating process. The two TMDs are depicted as half‐cylinders. The NBDs are shown as shorter half‐cylinders, which hold two molecules of ATP (grey‐shaded ovals) bound at their interface. The domain swap of TMDs and NBDs is indicated by a rotation of the TMDs relative to the NBDs. The membrane is symbolized by a wire frame box. The two outer gates are shown as colored disks. A functional outer gate is indicated in green, while a dysfunctional gate is shown in yellow. The two symmetry‐related rh123 binding modes are symbolized by dotted ovals (green lettering in normal or mirror writing). Arg + refers to binding‐mode selector residues introduced in symmetry‐related positions 132 (right) or 773 (left). An X indicates that the respective binding mode was deselected by introduction of the respective arginine residue and that the corresponding gate is not used (no arrow). (A) Wild‐type P‐gp. Depending on its binding mode, rh123 may exit the binding pocket via outer gate 1 (left) or 2 (right). (B) Mutant Y310A: Gate 1 is dysfunctional, a facilitative (defective gate 1) and active transport component (functional gate 2) are present simultaneously. (C) Mutant Y953A: Gate 2 is dysfunctional. Again a facilitative (defective gate 2) and active transport (functional gate 1) component superimpose. (D) Mutant Q132R: rh123 binds in mode 1 only and exits via gate 1. (E) Mutant Q773R: rh123 binds in mode 2 only and exits via gate 2. Both mutants show active transport characteristics.^8^ (F) Mutant Y310A/Q725L/Q132R: rh123 binds in mode 1, outer gate 1 is dysfunctional. The mutant shows facilitative behavior, as indicated by a bidirectional arrow. (G) Mutant Y953A/Q132R: rh123 binds in mode 1 and exits via a functional gate 1. Active transport characteristics are retained, as the dysfunctional gate 2 is not used. (H) Mutant Y310A/Q725L/Q773R: rh123 binds in mode 2. Active transport is observed, as dysfunctional gate 1 is not used. (I) Mutant Y953A/Q773R: P‐gp adopts facilitative behavior, as rh123 binds in mode 2 and gate 2 is dysfunctional. (J) the double Arg + mutant Q132R/Q773R does not show rh123 transport^8^

We show that residue Y310 in TM5 forms part of an outer gate, when rh123 binds in mode 1, while Y953 in TM11 forms part of a second outer gate, when rh123 binds in mode 2. Figure 6 outlines this concept and depicts the experimental outcome in schematic form. In panel A, two symmetry‐related outer gates, which provide alternative exit paths for rh123, are depicted. As mutation‐induced dysfunction of one outer gate still leaves the other outer gate intact (Figure 6B and C) we used transporter mutants in which rh123 was confined to bind in only one of the two possible modes (Figure 6D and E). The existence of two symmetry‐related outer gates would result in the following outcome of experiments: (a) In mutants which bind rh123 in mode 1 only, mutation‐induced dysfunction of outer gate 1 would lead to loss of active transport characteristics (as indicated by a bidirectional arrow) (Figure 6F). (b) When rh123 would again bind in mode 1, but now gate 2 would be rendered dysfunctional, active transport characteristics would be retained (Figure 6G). (c) When rh123 binding would be allowed in mode 2, mutation of gate 1 would not affect active transport characteristics (Figure 6H). (d) On the other hand, mode 2 binding of rh123 would again lead to a loss of active transport characteristics, when outer gate 2 is impaired (Figure 6I). We showed earlier that preventing binding of rh123 in either of the two modes abolishes transport in the Q132R/Q773R double mutant (Figure 6J).^8^ Thus, out of four mutants which combine outer gate mutations with selector mutations two would be predicted to lose active transport characteristics, while the other two would be predicted to retain them. This is exactly what was found experimentally. Retention of active transport characteristics in two of the four mutants clearly excludes the possibility that rh123 uses a single exit gate to leave the central binding cavity of the transporter.

It is important to note that the criterion for assessment of a dysfunctional outer gate is qualitative and not quantitative. Any mutation‐induced change in transport rates would therefore be irrelevant to the concept. Outer gate dysfunction in P‐gp mutants is experimentally demonstrated by the inability of these mutants to hold against a concentration gradient. As this finding could also result from complete transport deficiency of the mutants, the capacity for downhill transport of rh123 needed to be demonstrated. Indeed, higher transport rates were found in the two transporter mutants Y310A/Q725L/Q132R and Y953A/Q773R than in negative controls. The observation that TRQ inhibits rh123 efflux in these mutants proves that downhill transport is indeed P‐gp mediated. Taken together, the adoption of facilitative transport characteristics is thus a reflection of an outer gate dysfunction in an otherwise transport competent mutant of P‐gp.

Could alternative explanations for a loss of active transport characteristics in the Y310A/Q725L/Q132R and the Y953A/Q773R be provided? Outer gate mutations were introduced in the Q132R and the Q773R background. These mutants showed active transport with rh123 transport rates of 50 and 25% of wild‐type P‐gp, respectively.^8^ In the present study, the Y310A/Q725L/Q773R and the Y953A/Q132R mutants were shown to retain active transport characteristics, which requires NBD‐TMD coupling. These mutants contain the identical mutations found in the Y310A/Q725L/Q132R and the Y953A/Q773R mutants, but in reverse combination. Furthermore, basal ATPase and ATPase stimulation assays have been reported in the literature for the Y310A, Y953A, Q725C, and Q725A single mutants and modulation by different substrates and inhibitors has been shown. This supports the notion that domain coupling in these mutants is not compromised.^23^, ^24^, ^25^, ^26^

The gene encoding for human P‐glycoprotein has arisen from a monomeric ancestor by gene duplication and subsequent evolutionary divergence of the two halves.^27^ Nevertheless, sequences of the P‐gp N‐ and C‐terminus are similar and twofold rotational symmetry of the P‐gp structure is retained. Symmetry‐related structural elements in the transporter can thus serve analogous functions, as illustrated by symmetry‐related nucleotide^5^ and substrate binding.^7^, ^8^ Data presented in this manuscript establish the existence of two symmetry‐related outer gates. Therefore, the duplicate presence of functional elements is a consequence of protein architecture and a hallmark at several steps of the transport cycle and extends to the outer gates.

P‐gp spans the plasma membrane as a helical bundle. In the course of the transport cycle, rearrangements of transmembrane helices occur. As volume work against a surrounding membrane environment would not be favored energetically, these rearrangements need to occur with minimal changes in the cross‐sectional diameter of the protein. We propose that the existence of two distinct and functionally separated outer gates helps in keeping such changes small. Rather than requiring diametrical movements of protein domains, substrate translocation would rather involve rotational movements of protein subdomains, which would ensure functionality of the transporter within the confines of the physiological environment of a lipid bilayer.

Taken together our experiments clearly establish the existence of two outer gates, which operate contingent on the binding mode of the substrate rh123. Residue Y310 is involved in outer gate formation, when rh123 binds in mode 1, while symmetry‐related residue Y953 is involved, when substrate binds in mode 2. Positions of tyrosine residues Y310 and Y953 in CEM structures of human P‐gp^5^, ^6^ are in agreement with their biochemically demonstrated role in outer gate formation.

## 5 CONCLUSION

The present study demonstrates that the duality, which is observed for ATP and substrate binding of P‐glycoprotein, extends to the process of outer gating. Therefore, twofold rotational symmetry of the protein architecture is reflected functionally at several steps of the transport cycle. This needs to be given adequate consideration in studies addressing the functional biology of the transporter, its interaction with drugs, and for drug‐drug interactions at the level of the transporter.

## DISCLOSURE

The corresponding author on behalf of all authors declares that no competing interests exist.

## AUTHORS’ CONTRIBUTIONS

Participated in research design: Nasim, Chiba. Conducted experiments: Nasim, Schmid. Contributed new reagents or analytical tools: Schmid, Sohail. Performed data analysis: Nasim, Schmid, Chiba. Wrote or contributed to writing of the manuscript: Chiba, Nasim, Schmid, Sitte, Stockner, Szakács. This work was supported by the Austrian Science Fund (FWF) within the scope of “Spezialforschungsbereich” SFB35 (project part 3506 to HHS, 3509 to PC, 3524 to TS and 3526 to GS) and EU COST Action CM1306 to TS.
