# Supplementary material for: Active transport of rhodamine 123 by the human multidrug transporter P‐glycoprotein involves two independent outer gates
Source: Pharmacol Res Perspect. 2020 Mar 31;8(2):e00572. doi: 10.1002/prp2.572 (PMC7105846; doi:10.1002/prp2.572)
Supplement: Supplementary file 1 [file PRP2-8-e00572-s001.docx]

**Supplemental Data**

**Fig. S1:**

Time dependence of fractional filling of cells with rh123. Wild-type, mock transfected cells and mutants Y953A/Q132R, Y953A/Q773R, Y310A/Q725L/Q132R and Y310A/Q725L/Q773R are shown. Except for the two triple mutants Y310A/Q725L/Q132R, Y310A/Q725L/Q773R data points refer to mean ± standard deviation of at least three independently performed experiments. For the former, fractional filling is shown for up to 50 minutes. Here data points conform to averages of two independently performed experiments. In all cases steady-state levels were reached in less than 10 minutes.

**
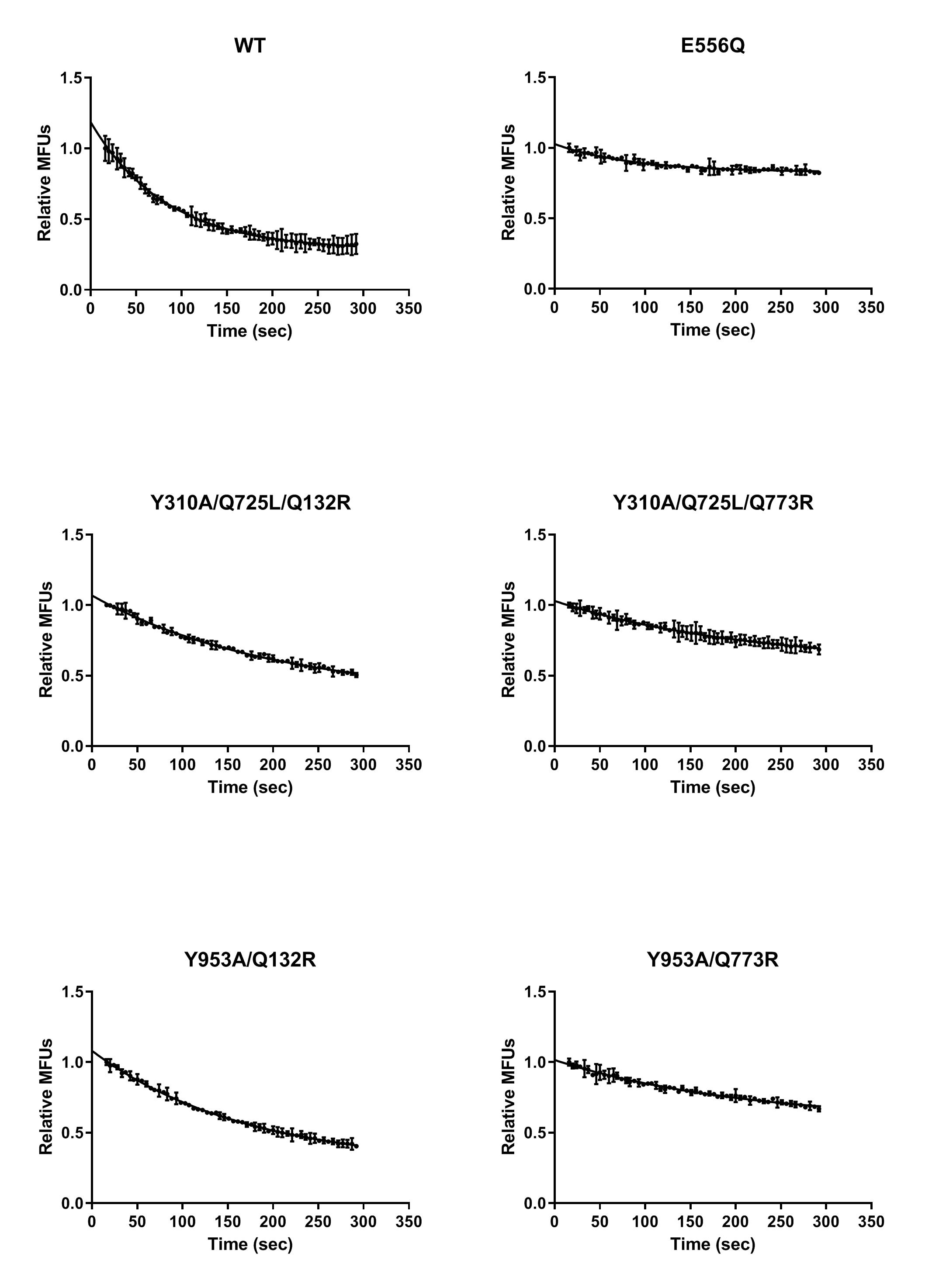
**

**Fig. S2:**

Efflux of rh123 over a time period of 5 minutes. Data for wild-type and mutants E556Q, Y310A/Q725L/Q132R, Y310A/Q725L/Q773R, Y953A/Q132R and Y953A/Q773R are shown. Data were acquired continuously. Data points represent the average of five second intervals and corresponding standard deviations. As within the first 20 to 30 seconds after resuspension of cell pellets in 37°C culture medium cells showed non-exponential efflux characteristics, these data points were excluded from the fit.


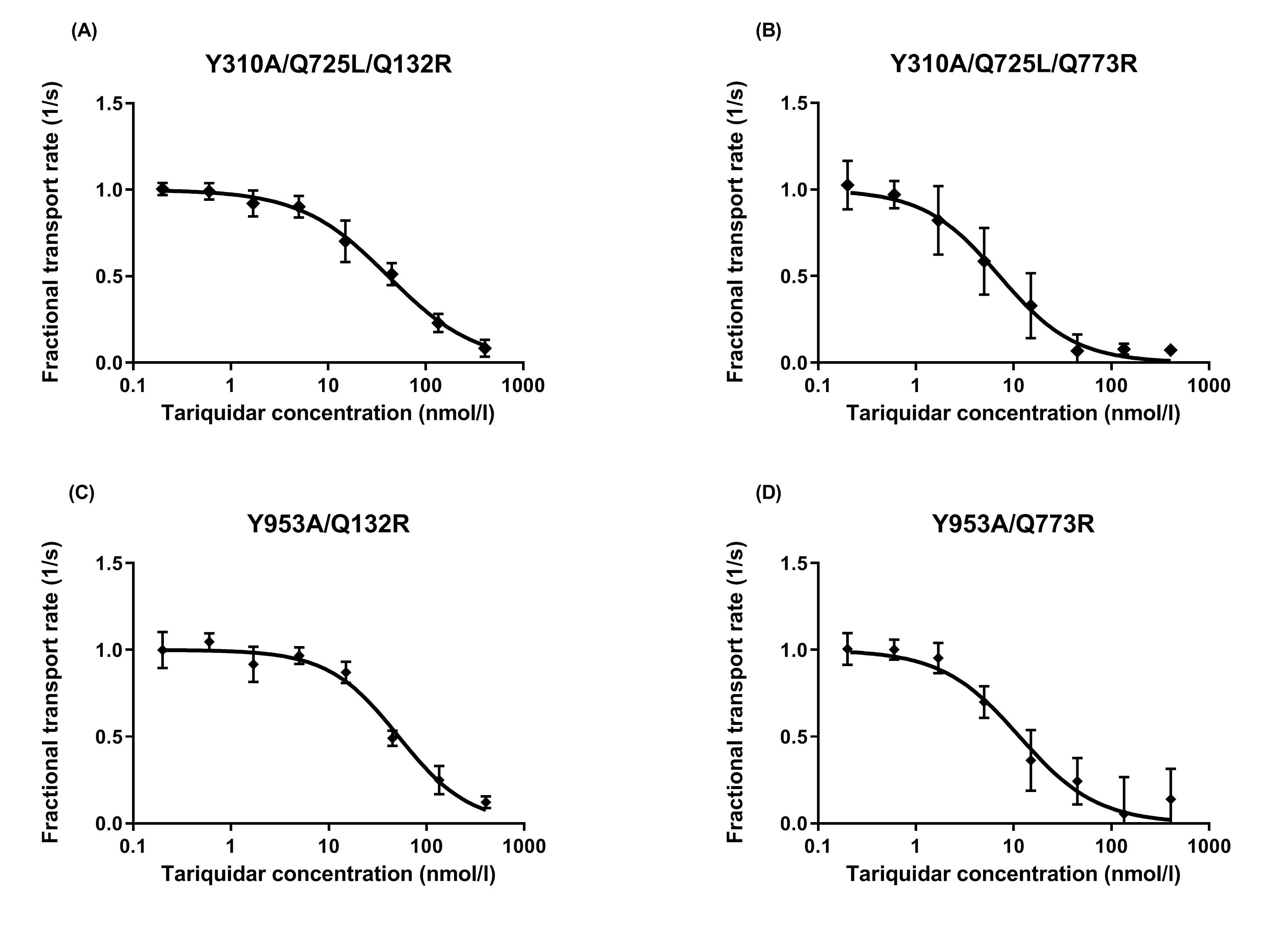


**Fig. S3:** Fractional zero-trans efflux rates of rh123 are shown as a function of the TRQ concentration for wild-type, the catalytic glutamate (negative control) and the four outer gate mutants in the binding mode selector background. Eight different concentrations of TRQ were prepared in a serial 1:3 dilution. A: mutant Y310A/Q725L/Q132R; B: mutant Y310A/Q725L/Q773R; C: mutant Y953A/Q132R and D: mutant Y953A/Q773R. Data points refer to the mean ± SD of at least three independent experiments performed in duplicate. Hyperbolic concentration response curves were fitted to the data points. IC_50_ values are shown in Table S1.

**Table S1:** IC_50_ values for tariquidar in wild-type and mutants

| **ABCB1** | **IC_50_ values (nM) 95% Cl** |
| --- | --- |
| **Wild type** | 3.0 - 4.4 |
| **Y310A** | 11.3 – 35.4 |
| **Y953A** | 22.2 – 221.0 |
| **Q132R** | 9.0 – 14.0 |
| **Q773R** | 2.5 - 9.7 |
| **Y310A/Q725L** | 22.0 – 87.2 |
| **Y953A/Q132R** | 39.0 – 69.3 |
| **Y953A/Q773R** | 8.0 – 17.3 |
| **Y310A/Q725L/Q132R** | 35.1 – 49.0 |
| **Y310A/Q725L/ Q773R** | 6.0 - 9.3 |
